# Comparing the Efficacy of Mindfulness-Based Stress Reduction Therapy with Emotion Regulation Treatment on Quality of Life and Symptoms of Irritable Bowel Syndrome 

**Published:** 2018-07

**Authors:** Fatemeh Ghandi, Amir Sadeghi, Maryam Bakhtyari, Saeed Imani, Saeed Abdi, Seyed Shahab Banihashem

**Affiliations:** 1 Gastroenterology and Liver Diseases Research Center, Research Institute for Gastroenterology and Liver Diseases, Shahid Beheshti University of Medicine Sciences, Tehran, Iran.; 2 Basic and Molecular Epidemiology of Gastroenterological Disorder Research Center, Research Institute for Gastroenterology and Liver Diseases, Shahid Beheshti University of Medicine Sciences, Tehran, Iran.; 3 Department of Clinical Psychology, Shahid Beheshi University of Medicine Sciences, Tehran, Iran.; 4 Department of Clinical and Health Psychology, Shahid Beheshti University, Tehran, Iran.; 5 Department of Psychiatry, Shahid Beheshti University of Medicine Sciences, Tehran, Iran.

**Keywords:** *Emotion Regulation*, *Irritable Bowel Syndrome*, *Mindfulness-Based Stress Reduction*, *Quality of Life*

## Abstract

**Objective:** Irritable bowel syndrome is a common gastrointestinal disorder. The perception of stress and GI-speciﬁc anxiety play a key role in irritable bowel syndrome (IBS). The present study aims at comparing the efficacy of mindfulness-based stress reduction therapy with emotion regulation on the quality of life and severity in patients IBS.

**Method** **:** This randomized clinical trial was conducted in 3 phases: pretest, posttest, and follow-up. Follow-up was performed 2 months after the last intervention. The study population consisted of 24 IBS patients who were randomly selected according to Rome-IV Criteria and were then divided into 3 eight-member groups: (1) mindfulness-based stress reduction, (2) emotion regulation, and (3) control group. IBS-QOL34 and IBS-SSS were administered as assessment tools to all the 3 groups. The experimental groups were subjected to MBSR and ER psychotherapy, while the control group received no psychological intervention. After the 2-month follow-up, the 3 groups were evaluated again.

Results: The results revealed that MBSR improved the quality of life of IBS patients and dicreased severity of their condition. The findings of between and within subjects design revealed that the difference between MBSR and control groups was significant in IBS at follow-up (p = 0.01).

**Conclusion: **MBSR could be considered as a new, effective, and stable method in psychotherapy, in irritable bowel syndrome.

Irritable bowel syndrome is a functional bowel disorder with symptoms, including abdominal pain and disturbed bowel movements (1, 2). While the disease is experienced by 10% of the adult population of the United States (14% of women and 8% of men) (3), the prevalence rate of IBS in Iran has been reported to be 5.8% in the general population and 3% to18.4% in specific Iranian populations; as a result of IBS, patients’ quality of life is impaired and high costs are incurred on the health care system (4). 

The most common health care provided for the irritable bowel syndrome focuses on diet and lifestyle management (5), and pursuant medical treatments often incur high costs and side effects, however, they may only eliminate the symptoms temporarily, not the disease (6, 7). In general, there are no medical or drug regimen options to manage the wide range of IBS symptoms, and Attention is shifted to the biopsychosocial factors (8) due to the lack of effective medication and medical treatment to relieve IBS symptoms.

Cognitive information and external stressors are able to influence the senses, movement, and secretion of the digestive tract through neural connections (9). As a result, muscle contractions, GI secretion, and pain can be increased due to mental stress and negative interpretation of emotions (10). The physiology of the digestive system, the experience of mental health symptoms, and their treatment have impact on the treatment outcome. Stress and emotions may start neuroendocrine activity through the brain-gut axis, and as a result, the stomach, endocrine glands, and immune system function effectively (11).

IBS patients have high levels of GI-specific anxiety, while it has been observed that it is acted as an inside stressor, which might be continued steadily by IBS symptoms even in the absence of external stressors (12). Psychotherapies, such as cognitive-behavioral therapy (CBT) and hypnosis, have exhibited positive effects on improving symptoms, and the results can reflect the importance of the biopsychosocial aspects of IBS (13). Both past and prospective studies have shown that chronic stress, acute stress, and increase in perceived stress tend to exacerbate IBS symptoms (14).

As psychosomatic medicine deals with physical illness in terms of emotional disorders, pharmacological and non-pharmacological treatments seem necessary to alleviate and eliminate these patients’ emotional problems. Moreover, some therapeutic solutions can reduce the negative consequences of the disease and improve patients’ quality of life. Therapeutic approaches for patients who have difficulty regulating emotions must include treatments that are able to increase internal pressure and emotional awareness and regulate emotional arousal modes through the process of understanding. A deep understanding of the nature of emotional disorders reveals that commonalities of this disorder distinguish between its etiology and hidden structures (15).

Psychological treatment of IBS symptoms has been taken into consideration due to the increase in the association of response to stress with the events related to visceral tracts, the weak or inappropriate dealings with these events, compliance with the disease, and limitations in the medical treatment. Accordingly, the symptoms of irritable bowel syndrome are associated with such factors as emotional and affection arousal, reduction of stress, and increase in positive personal moods; thus, mind and body interventions, such as MBSR and ER, can break the physiological arousal cycle and physiological symptoms (8).

Such an intervention is aimed at comparing the effect of treatment in each of these therapeutic methods. This was the first study on patients suffering from irritable bowel syndrome in comparison with a control group.

## Materials and Methods

Patients who referred to Gastroenterology and Liver Diseases Research Center of Shahid-Beheshti University of Medical Sciences, Tehran, and were diagnosed with IBS based on Rome-IV Criteria, participated in this study. Inclusion criteria of the study were as follow: age 18 to 45; having a high school diploma as the minimum educational requirement, (as a certain level of motivation and ability in identifying intellectual and emotional states is required for active participation in psychotherapy); and absence of psychosis, neurological disorder, and substance abuse. Exclusion criteria included lack of interest in continuing the treatment and missing more than 2 sessions.

Eligible participants were 45 patients who were randomly placed into three 15-member groups. All patients received medication and 1 group was treated with eight 90-minute sessions of MBSR group therapy, the other group was treated with eight 90-minute sessions of group therapy based on emotional regulation, while the third group was the control group who received only medical therapy. Both treatments were administered by a psychotherapist. IBS-QOL34 and IBS-SSS scales were used for all the 3 groups. After the 2-month follow-up, 3 groups were evaluated again.

Data were analyzed using SPSS software Version 22. The employed statistical methods included descriptive statistics and analysis of covariance (ANOVA) as per between- and within-subjects design.

***Psychological Questionnaire Measures***

***Quality of Life Questionnaire, Especially in Patients with IBS (IBS-QOL-34)***

Quality of Life Questionnaire for IBS patients was designed by Patrich and Drossman in 1998 and was used to assess the quality of life in these patients (yielding a total internal consistency scale of 0.094). This scale consists of 34 questions and 8 subscales: dysphoria, social reaction, health worries, body image, interpersonal relationships, food avoidance, sexual concerns, and interferences with daily activities. Each question is scored on a 5-stage Likert scale (5). The Persian version of the questionnaire has appropriate diagnostic validity, and subscales of this questionnaire, as well as the overall quality of life scale, have relatively acceptable internal consistency coefficients. The reported Cronbach's alpha coefficient for the subscales are as follow: dysphoria 0.88, interferences with daily activities 0.67, body image 0.72, health worries 0.57, food avoidance 0.52, social reaction 0.71, sexual concerns 0.76, and interpersonal relationships 0.93 (16).

***Questionnaire of Severity of Bowel Symptoms (IBS-SSS)***

IBS Severity Scale is a 5- item tool used as a visual scale analog (VSA) to measure the severity of abdominal pain, abdominal pain frequency, severity, abdominal distension, and discontent with IBS bowel habits and its impact on quality of life. Scores of all the items are summed together to obtain the IBS severity. The total score of the tool ranges from 0 to 50, with a higher score indicating higher intensity of IBS. This test has been used in many clinical trials and its changes are significantly associated with changes in quality of life, anxiety, and depression scores. Cronbach’s alpha for internal consistency of the scale is reported to be 0.69 (10). The Persian version of this questionnaire has such an excellent scale reliability and validity that the overall scale score is correlated with the total score of quality of life (0.62) and anxiety depression (0.45). Cronbach's alpha of the scale is reported to be 0.68 (17).

***Intervention***

**Figure1:**
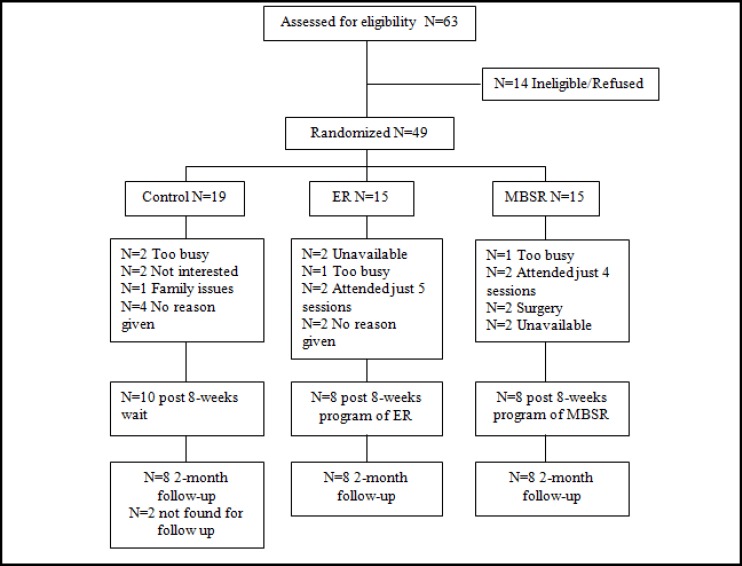
Overall Design and Subject Flow

Mindfulness-based therapy is designed for IBS patients based on the mindfulness-based stress reduction (MBSR). This therapy is a multi-component approach that uses mindfulness skills, such as knowledge over breathing process, conscious eating, and other daily activities, leading to reductions in IBS symptoms. In this approach, people learn to observe and experience pain, thoughts, and feelings without any judgment. When patients focus on the present moment experience, it is possible to accept pain as a feeling for which they have no definition. This way, people may understand and experience thoughts and feelings for which they have no definition and, therefore, they can be less controlled by pain, anxiety, and depression (18)(Figure 1).

According to the literature review, mindfulness is performed with targeted intervention on cognitive coping skills, such as catastrophizing, which leads to exaggerated symptoms of IBS (19). With respect to the effect of MBSR mechanism on gastrointestinal signs and IBS symptoms, it can be claimed that MBSR can increase the capacity and capability of information processing system (20).

Also, the role of emotion regulation disorders in the spread and persistence of psychosomatic diseases, such as functional gastrointestinal disorders, has also been examined. Previous studies have shown that IBS patients have difficulty regulating their emotions (21). Emotion regulation is considered as an intermediary coping strategy in the context of adaptation to stress, and those who use maladaptive cognitive coping strategies experience stress more than those who use adaptive strategies. Maladaptive strategies, catastrophizing, and rumination in dealing with stress lead to increased emotional problems and, therefore, influence some of the features of emotion regulation and all levels of stress; cognitive emotion regulation helps people manage their emotions after experiencing a stressful event (21).

Effective emotional regulation requires skills in such areas as "emotional awareness" and "acceptance". According to this definition, when a person uses maladaptive strategies and faces difficult emotional experiences, he/she is unable to control the experienced emotions for being involved in the control of a goal-oriented behavior and prevents the experienced excitement to move in its direction (22).

Recent cognitive science researches have shown that mindfulness meditation alters the activity of neural circuits involved in the visceral nerve awareness and sensitivity to internal stimuli and regulates them (23). In addition, mindfulness is effective in improving the ability to control attention processes (24).

Psychotherapy is based on the emotional regulation in which emotional processes are the main targets of treatment (25). The effectiveness of this treatment on anxiety disorders, depression, and other disorders that have strong emotional components has been studied (26). However, its effect in psychosomatic diseases has not yet been assessed.

## Results

The study population included 24 patients, consisting of 13 females (54.2%) and 11 males (45.8%). Of the patients, 15 (62.5%) were single and 9 (37.5%) married. Nine patients (37.5%) had high school diploma, 12(50%) bachelor’s degree, and 3 (12.5%) master’s degree (Table 1). The mean and SD of each variable are reported in Table 2. 

In the present study, analysis of covariance (ANCOVA) was used to control the effect of pretest scores and the treatment effect study on the posttest as dependent variables. The analysis of between- and within-subjects design was also applied to the study at the follow-up stage. The Levin test was used to assess the equality of variances; the obtained results confirmed the equality of variances across variables, so these results suggested the use of ANCOVA for data analysis. Quality of life scores between the 2 groups (MBSR and Control) at pretest and posttest revealed that quality of life scores in the posttest increased only in MBSR group. IBS severity scores between the 2 groups (MBSR and Control) at pretest and posttest revealed that IBS severity scores in the posttest increased only in MBSR group (Tables 2 and 3). 

Quality of Life and IBS Severity Scales posttest scores of the 2 groups of MBSR and control were improved, and pretest scores proved to be significant (p≤0.05) (Table 3).

The scales of quality of life and IBS severity between the 2 groups of mindfulness-based stress reduction and control for the effects of pretest scores revealed a significant difference between emotion regulation and control groups, and MBSR and control groups on IBS severity (p≤0.01) (Tables 4,5 and 6).

## Discussion

The present study aimed at comparing the efficacy of mindfulness-based stress reduction with that of psychotherapy-based emotion regulation on quality of life and symptoms of IBS. According to the obtained results, MBSR has improved quality of life and reduced symptoms of IBS in patients suffering from IBS. The findings also revealed that the difference between MBSR and control groups, and between ER and control groups was significant for IBS severity at follow-up.

The obtained results revealed a significant difference between MBSR and control groups on IBS severity at follow-up and improved quality of life. Previous studies were conducted on the positive outcomes of mindfulness-based stress management intervention (27). Evidence shows that mindfulness is related to mental health indexes, such as high levels of positive effects, liveliness, adaptive emotional regulation, lower levels of negative effects, and psychological symptoms. On the other hand, mindfulness-based techniques lead to reduced stress and pain symptoms. Mindfulness-based interventions lead to reduced frequency of negative automatic thoughts and ultimately to psychological welfare. Mindfulness-based interventions lead to metacognitive awareness and reduction in mental rumination and negative thoughts (28). 

Furthermore, in this study, no significant difference was observed in the emotion regulation group in quality of life; this finding can be justified by the notion that the quality of life of patients with IBS has been defined according to some limitations of the patients; quality of life in IBS is a multi-component index, including social relations, job satisfaction or education, sexual activity, and mental conditions. These patients have limited social relationships and suffer from different symptoms of depression and have many concerns about the nature of their disease. Thus, improving the quality of life requires addressing all these problems (29).

Treatment through emotion regulation can be beneficial for improving quality of life and reducing other variables in patients with IBS because of its simultaneous emphasis on emotional insight and clear behavioral guidelines. However, in this study, at the posttest stage of emotion regulation group, this treatment had no significant effect on reducing IBS severity. It seems that to increase the effectiveness of treatment, it should be administered for a longer duration. On the other hand, improvement in IBS disease severity takes more time to obtain more emotional tolerance to deal with more difficult emotional experiences.

The technique of observing one’s experience with equanimity rather than attempting to alter or control one’s experience is central to mindfulness-based stress reduction; yet, mindfulness as a form of emotion regulation, is in many ways foreign to the framing of emotion regulation in conventional scientific literature (30). Traditionally, emotion regulation has been cast in terms of 2 major strategies: “suppression” and “reappraisal”. Suppression attempts to limit or exaggerate the representation of emotion itself, whereas reappraisal strategies seek to alter the context in which an emotion-inducing stimulus is viewed, thereby altering the emotion provoked. Unlike effortful suppression or reappraisal strategies, mindfulness based cognitive therapy does not seek to achieve an idealized, non-aversive goal state, but rather it attempts to create a psychological distance between the emotion and the individual, limiting its behavioral consequences. Establishing psychological distance from aversive emotions may be a part of the reappraisal process, but mindfulness differs from such processes in that it treats the labeling or monitoring of the experience as an end in it-self rather than a means by which to control the emotion (30).

Treatment of MBSR includes a focus on all emotions instead of suppressing them or avoiding emotional events. Bayer et al. studied and compared the relationship between mindfulness-based treatment and emotion regulation in reducing experimental avoidance. Their findings showed that mindfulness has a more opposite relationship with emotion regulation problems, such as techniques for understanding and accepting problems and using emotion regulation in comparison with emotion regulation. Also, mindfulness-based treatment showed a more opposite relationship with experimental avoidance through emotion regulation (32). As indicated by evidence, mindfulness is linked to an increase in metacognitive awareness. The capability to experience thoughts and emotions through a focused approach in which the thoughts and emotions are experienced as mental events and not as an exact reflection of reality. As a result, increased trainings on attention and metacognitive awareness lead to changes in tactics employed against internal negative experience through increased acceptance of thoughts and emotions (30). 

## Limitation

This study had several limitations. One limitation was that the type of medicine and diet was not statistically controlled. Also, small sample size, non-simultaneous and non-parallel administration of treatment to the 2 respective groups, and administration of both therapeutic approaches by 1 therapist were other limitations of the study. Thus, for future studies, controlling medicines and increasing the size of the control group are suggested, and it is also recommended that each therapeutic method be implemented by 1 therapist separately and simultaneously.

## Conclusion

In comparison with emotion regulation treatment, mindfulness group therapy, which is based on stress reduction, has a greater effect on the quality of life, mindfulness components, anxiety, experimental avoidance, and reduced irritable bowel syndrome symptoms in the long- and short- run. The results obtained by the present study on the efficacy of mindfulness-based group therapy based on stress reduction are in agreement with those obtained by previous studies. Such efficacy is justifiable from various approaches. On the other hand, the long- and short-term efficacy of emotion regulation-based psychotherapy did not conform to the results obtained by previous studies. Also, the inefficacy of the latter approach on quality of life and factors influencing its efficacy need to be further investigated in future studies with diverse populations.

**Table1:** Contents of Group Therapy Sessions

**Interventions**	**Sessions**	**Content**	**Sessions Implementation**	**Observation and ** **Implementation**
MBSR	1	Mindfulness during daily routines, Physical Scan Exercise	Assessment of life quality and disease severity	
	2	Mindfulness Meditation, Walking on the street exercise, surfing on negative emotions, mountain meditation	Assessment of body scanning exercise	Clinical psychologist
	3	Awareness of what we hear, breathing space exercise (for stressful situations)		Clinical psychologist
	4	Meditation focused on emotions and thoughts, breathing space exercise	Breathing space exercise in groups of two	
	5	Meditation in seating position while focusing on sounds, breathing, emotions, thoughts, stretching conscious exercises		Clinical psychologist
	6	Meditation in seating position while focusing on thoughts, an explanation on disease lapse cycle	Breathing space exercise	
	7	Meditation in seating position and breathing space		Clinical psychology
	8	Physical Scanning and Meditation(31)	Assessment of life quality and disease severity	
ER	1	Acquaintance with the disease, introduction to and identifying emotions	Assessment of life quality and disease severity Emotional gradation	Clinical psychology
	2	Identifying emotions in situations, thoughts, emotions and the body	Assessment of emotions aimed at emotional vulnerability	
	3	Self-regulation, emotional solutions		
	4	Changing situations and attentions, re- assessment		Clinical psychology
	5	Not avoiding negative emotions and emotion regulation	Reassessment technique exercise	
	6	Exposure to negative emotions, emotional release, and calming	Emotional expression skills	Clinical psychology
	7 & 8	Emotion regulation technique(30)	Gradation of emotion regulation skills Assessment of life quality and disease severity	
Control	1 & 2	Assessment of life quality and disease severity		

**Table 2:** Demographic Characteristics of the Study Population in The MBSR, ER and Control Groups

**Variable**	**Category**	**MBSR**		**ER**		**Control**		**Total**	
	**Definition of Statistics**	**Frequency**	**Percent**	**Frequency**	**Percent**	**Frequency**	**Percent**	**Frequency**	**Percent**
Sex	1	3	12.5	4	16.7	4	16.7	11	45.8
	2	5	20.8	4	16.27	4	16.7	13	54.2
Marriage	1	4	16.7	7	29.2	4	16.7	15	62.5
	2	4	16.7	1	4.2	4	16.7	9	37.5
Education	1	3	12.5	3	12.5	3	12.5	9	37.5
	2	4	16.7	4	16.7	4	16.7	12	50
	3	1	4.2	1	4.2	1	4.2	3	12.5
Total		24	33.3	24	33.3	24		33.3	100

**Table 3:** The Descriptive Information of the IBS* Severity and Quality of Life of the Experimental Groups

**Variable**			**Pretest**		**Posttest**		**Follow-up**	
	**Category**	**Number**	**Mean**	**SD**	**Mean**	**SD**	**Mean**	**SD**
IBS	MBSR	8	29.37	4.749	10.87	3.09	9.37	4.68
	ER	8	32.25	8.82	15.50	6.02	16.62	7.36
	Control	8	26.25	9.85	20.12	10.43	24.75	8.46
Quality of Life	MBSR	8	74.50	20.94	55.62	11.10	52.87	16.14
	ER	8	99.62	30.39	81.25	28.23	67.25	25.44
	Control	8	75.25	29.85	65.00	25.65	74.50	32.09

* Irritable Bowel Syndrome

**Table 4:** Studying the Durability of Interventions by Comparing the Results of Posttest Follow-up in the Study Groups

**Variable**		**Sum of Squares**	**Df**	**Mean Square**	**F**	**P Value**
IBS	Levels	0.058	1	0.058	0.002	0.963
	Group Stages	51.440	2	25.720	0.958	0.401
	Groups	1420.243	2	710.121	13.237	0.01
Quality of Life	Levels	80.475	1	80.475	0.841	0.38
	Group Stages	1148.144	2	574.072	6.000	0.01
	Groups	4196.593	2	2098.296	5.774	0.05

**Table 5:** Comparing the 2 Groups Using Bonferroni Test in the Quality of Life and the IBS* Severity Variables

**Variable**	**Comparison Groups**		**Mean Differences**	**P value**
IBS	MBSR	ER	3.199-	0.94
		Control	10.80-	0.05
	ER	Control	7.60-	0.09
Quality of Life	MBSR	ER	13.18-	0.23
	ER	Control	18.19-	0.05
		Control	5.01-	0.99

* Irritable Bowel Syndrome

**Table 6:** Comparing Quality of Life and IBS Variables the MBSR and ER Groups Using Bonferroni Test in the Follow-up

**Variable**			**Mean Differences**	**P Value**
IBS	MBSR	ER	4.66-	0.26
		Control	13.69-	0.01
	ER	Control	9.02-	0.01
